# Bioengineered airway epithelial grafts with mucociliary function based on collagen IV- and laminin-containing extracellular matrix scaffolds

**DOI:** 10.1183/13993003.01200-2019

**Published:** 2020-06-18

**Authors:** Nick J.I. Hamilton, Dani Do Hyang Lee, Kate H.C. Gowers, Colin R. Butler, Elizabeth F. Maughan, Benjamin Jevans, Jessica C. Orr, Conor J. McCann, Alan J. Burns, Sheila MacNeil, Martin A. Birchall, Christopher O'Callaghan, Robert E. Hynds, Sam M. Janes

**Affiliations:** 1 Lungs for Living Research Centre, UCL Respiratory, University College London, London, UK; 2 UCL Ear Institute, The Royal National Throat Nose and Ear Hospital, London, UK; 3 Respiratory, Critical Care and Anaesthesia, UCL Great Ormond Street Institute of Child Health, London, UK; 4 Stem Cell and Regenerative Medicine, Birth Defects Research Centre, UCL Great Ormond Street Institute of Child Health, London, UK; 5 Dept of Materials and Science Engineering, The Kroto Research Institute, North Campus, University of Sheffield, Sheffield, UK; 6 Nick J.I. Hamilton and Sam M. Janes are joint-senior authors

## Abstract

Current methods to replace damaged upper airway epithelium with exogenous cells are limited. Existing strategies use grafts that lack mucociliary function, leading to infection and the retention of secretions and keratin debris. Strategies that regenerate airway epithelium with mucociliary function are clearly desirable and would enable new treatments for complex airway disease.

Here, we investigated the influence of the extracellular matrix (ECM) on airway epithelial cell adherence, proliferation and mucociliary function in the context of bioengineered mucosal grafts. *In vitro*, primary human bronchial epithelial cells (HBECs) adhered most readily to collagen IV. Biological, biomimetic and synthetic scaffolds were compared in terms of their ECM protein content and airway epithelial cell adherence.

Collagen IV and laminin were preserved on the surface of decellularised dermis and epithelial cell attachment to decellularised dermis was greater than to the biomimetic or synthetic alternatives tested. Blocking epithelial integrin α2 led to decreased adherence to collagen IV and to decellularised dermis scaffolds. At air–liquid interface (ALI), bronchial epithelial cells cultured on decellularised dermis scaffolds formed a differentiated respiratory epithelium with mucociliary function. Using *in vivo* chick chorioallantoic membrane (CAM), rabbit airway and immunocompromised mouse models, we showed short-term preservation of the cell layer following transplantation.

Our results demonstrate the feasibility of generating HBEC grafts on clinically applicable decellularised dermis scaffolds and identify matrix proteins and integrins important for this process. The long-term survivability of pre-differentiated epithelia and the relative merits of this approach against transplanting basal cells should be assessed further in pre-clinical airway transplantation models.

## Abstract 2

**Collagen IV- and laminin-rich decellularised dermis scaffolds support a mucociliary airway epithelial graft but *in vivo* transplantation in pre-clinical models is challenging** http://bit.ly/2IdQp5d

## Introduction

The respiratory mucosa lines the internal surface of the trachea and bronchi and consists of a pseudostratified, multiciliated epithelium containing mucus-secreting goblet cells [1]. The respiratory mucosa performs a vital array of functions, including acting as a barrier against infection and clearing secretions from the lower airways *via* the mucociliary escalator [2, 3]. Existing methods to restore respiratory mucosa following airway reconstruction and cancer resection rely on the transfer of muscle on a vascularised pedicle and skin grafting. Whilst these can re-epithelialise small sections of airway, they are not suitable for reconstruction of larger areas as the epithelium retains stratified squamous histology and thus lacks the ciliated and mucosecretory cells required for normal functionality [4]. The epidermis also has a higher rate of epithelial turnover than respiratory epithelium, which may contribute to airway sloughing and obstruction in these patients [5]. Buccal epithelium has been used in mucosal grafts and successfully applied to restore small sections of tracheal mucosa [6]; however, due to limitations in the extent of donor tissue that can be harvested, this approach is also not suitable for extensive proximal airway repair.

The ability to regenerate a transplantable respiratory mucosal layer with mucociliary function would be a significant step forward in the field of airway regenerative medicine. It would enable new therapies to treat long-segment mucosal diseases of the upper airways, including complex scarring and granulomatous conditions. Such a technique would also be highly relevant to gene editing approaches to treat genetic disorders such as cystic fibrosis, where cell engraftment poses a major challenge [7]. Examples of bioengineered tracheal replacements have been limited by slow mucosalisation following implantation [8–10] and bioengineered respiratory mucosal grafts might improve the safety and efficacy of such procedures.

Current reports of *in vitro* bioengineered upper airway mucosa have mainly focused on regenerating the mucosal layer on tracheal scaffolds [11, 12]. However, the *in vivo* application of these techniques is limited by the time taken for revascularisation to occur following transplantation. To overcome this, we envisage the use of a two-stage procedure [13] whereby a mucosal layer composed of respiratory cells (rather than cells from other epithelia, *e.g.* buccal [14, 15]) is generated *in vitro* and can be used to re-epithelialise a pre-vascularised implanted airway scaffold or be grafted directly onto the airway to replace damaged mucosa. This methodology more closely follows the principles of free tissue transfer, where well-vascularised graft beds are essential for successful outcomes [16].

In formulating a method to regenerate respiratory mucosa, careful consideration needs to be given to the extracellular matrix (ECM) environment. The ECM is a complex network of macromolecular proteins that are bound by specific cation-dependent cell surface receptors, the integrins, on the basolateral surface of epithelial cells [17]. Integrin–ECM binding leads to cascades of intracellular signalling that influence multiple cellular processes including attachment, proliferation, polarity and programmed cell death [18]. Evidence from investigations of the ECM in stratified epithelia, along with proteomic data examining the composition of the upper airway basement membrane, indicate that collagen I, collagen IV, laminin, vitronectin and fibronectin play important roles in modifying epithelial cell behaviour [19–21]. Here, the effect of these ECM proteins on respiratory epithelial cell attachment, expansion and differentiation *in vitro* was investigated with a view to optimising the ECM environment for bioengineered airway mucosa.

## Materials and methods

### Primary cell culture

Primary human bronchial epithelial cells (HBECs) were isolated from endobronchial biopsies from the human adult upper airways or from the bronchi of patients undergoing lobectomy (supplementary table S1). Ethical approval was obtained from a Research Ethics Committee (REC references: 06/Q0505 and 11/LO/1522). HBECs were maintained in bronchial epithelial growth medium (BEGM) (Lonza, Slough, UK) for attachment and proliferation experiments. For differentiation experiments, HBECs that had been isolated and maintained on mitomycin C-treated 3T3-J2 feeder layers with 5 µM ROCK inhibitor Y-27632 (3T3+Y) (Enzo Life Sciences, Exeter, UK) were used as previously described [22–24]. Cells isolated and expanded in BEGM were used between passages one to three, while cells isolated and expanded in 3T3+Y were used between passages one to six. Primary human lung fibroblasts were maintained in Dulbecco's Modified Eagle Medium (DMEM) (Gibco, Hemel Hempstead, UK) containing 10% foetal bovine serum (FBS) and were used no later than passage ten.

### Coating of tissue culture plastic with extracellular matrix protein

ECM proteins were used to coat tissue culture plastic using the recommended manufacturer's concentrations and a value above (high) and below (low) that concentration (medium). The ECM proteins used were collagen I from human neo-natal fibroblasts (Advanced Biomatrix, San Diego, USA) at 38.7 µg·mL^−1^, 387 µg·mL^−1^ and 968 µg·mL^−1^; collagen IV from human placenta (C8374, Sigma-Aldrich, Dorset, UK) at 10 µg·mL^−1^, 250 µg·mL^−1^ and 500 µg·mL^−1^; vitronectin from human plasma (V8379, Sigma-Aldrich) at 0.5 µg·mL^−1^, 1.25 µg·mL^−1^ and 2.5 µg·mL^−1^; laminin from human placenta (L6274, Sigma-Aldrich) at 1 µg·mL^−1^, 2 µg·mL^−1^ and 4 µg·mL^−1^; and 0.1% fibronectin from human plasma (F0895, Sigma-Aldrich) at 1 µg·mL^−1^, 25 µg·mL^−1^ and 250 µg·mL^−1^. Non-adherent 96-well plates (10 554 961, Fisher Scientific, Hemel Hempstead, UK) were coated with ECM proteins following the relevant manufacturer's protocol.

### Integrin blocking experiments

Antibodies previously reported to have blocking function against integrin subunits [25–32] were added at a concentration of 1:200 in BEGM and the antibody–cell suspension was kept on ice for 20 min before addition to ECM-coated or decellularised dermis scaffold containing wells for experiments examining cell attachment. Untreated cells were used as controls. In cell expansion experiments, cells were allowed to adhere for 2 h before they were washed with phosphate-buffered saline (PBS) and the medium replaced with BEGM containing the blocking antibodies. For experiments examining differentiation, blocking antibodies were refreshed with medium changes following air lift as indicated in figure legends. The antibodies used to block integrin subunits were: α1 (5E8D9; NBP2-29757, Novus Biological, USA), α2 (P1E6-C5; 359 304, Biolegend, San Diego, USA), α3 (ASC-1; MAB2056Z, Merck Millipore, Watford, UK), αv (NKI-M9; 32 7904, Biolegend), α5 (NKI-SAM-1; 328 004, Biolegend), α6 (GoH3; 313 614, Biolegend), α9β1 (Y9-A2; OBT1715Z, Bio-Rad, California, USA) and β1 (P5D2; MAB1959Z, Merck Millipore).

### Attachment, proliferation and metabolic assays

For assays involving ECM-coated plates, non-adherent 96-well plates were coated with ECM as described above and cells were seeded in BEGM before 30 min incubation at 37 °C, 5% carbon dioxide. Wells were washed three times with PBS to remove unbound cells and plates were frozen at −80 °C. To quantify the number of cells attached, plates were thawed to room temperature and a cell lysis buffer containing green fluorescent DNA dye (CyQUANT GR, Life Technologies, Hemel Hempstead, UK) was added according to the manufacturer's protocol. Fluorescence was measured using a microplate reader (excitation 480 nm, emission 520 nm) and background fluorescence was subtracted from these readings. To determine cell expansion on ECM substrates, the above steps were performed at 2 h and 48 h post-cell seeding. Cell number was calculated using a reference standard curve of known cell number.

To assess HBEC attachment to candidate scaffold materials, cells were seeded in non-adherent 96-well plates containing the scaffolds. After 1 h, the wells were washed four times with PBS to remove unbound cells and trypsin was applied to detach the adhered HBECs. Scaffolds were assessed by light microscopy to ensure detachment of adhered cells, collected and plated into a separate 96-well plate. A DNA dye (CyQUANT GR, Life Technologies) was used to compare cell attachment as described above. To assess the viability of epithelial cells seeded on decellularised dermis or tracheal scaffolds, a cell viability assay (alamarBlue, Life Technologies) was used. Cells were seeded onto decellularised scaffolds at the bottom of a 96-well plate and incubated for 1 h. Scaffolds were then washed with PBS, transferred to a new well and alamarBlue cell viability reagent was added in medium (1:10 ratio). After 2 h, fluorescence (excitation 560 nm, emission 590 nm) was recorded and a final value calculated by subtracting values generated from negative control wells containing scaffolds but no epithelial cells.

### Air–liquid interface culture and trans-epithelial electrical resistance

For standard air–liquid interface (ALI) cultures on positron emission tomography (PET) membranes, HBECs (1×10^6^) were seeded in 0.4 μM, 12 mm PET membrane transwells (Corning, Flintshire, UK) and incubated at 37 °C with 5% carbon dioxide supply [33]. ALI conditions in which medium was removed from the apical well were applied after 2 days. Cells were fed with ALI medium containing BEGM and DMEM at a ratio of 1:1 supplemented with 100 μg·mL^−1^ streptomycin and 100 units·mL^−1^ penicillin, as well as freshly prepared retinoic acid equivalent to 100 nM final concentration. Trans-epithelial electrical resistance (TEER) was measured using an EVOM2 resistance meter and Endohm chamber (World Precision Instruments, USA). Both the apical and basolateral sides of cultures were filled with BEBM (Lonza) and three replicate readings were taken for each well, allowing values to stabilise for 5–10 s each time.

### Production of candidate scaffolds

To produce decellularised dermis, glycerol-preserved cadaveric human skin was sourced from the Euro Tissue Bank (Beverwijk, The Netherlands). Decellularisation was carried out by washing three times in PBS over a 48-hour period and incubating the skin for 24 h at 37 °C in 1M sodium chloride as described by Deshpande *et al.* [34]. The epidermis was then peeled off and the decellularised dermis was used either directly or stored for up to 1 month at 4 °C in DMEM. POSS-PCU was provided by the Department of Nanotechnology (University College London, UK), where it was produced using previously published methods [35]. Biomimetic collagen I scaffolds were prepared using custom-made absorbers following the manufacturer's protocol (RAFT™, Lonza). In brief, rat tail collagen I (First Link, Wolverhampton, UK), DMEM and minimum essential medium (MEM) 10x (ThermoFisher Scientific, Hemel Hempstead, UK) were combined in an 8:1:1 ratio, neutralised with 5M sodium hydroxide and incubated for 15 min to allow the solution to gel. Absorbers were placed onto the surface of each well for 15 min to dehydrate the gel and produce translucent collagen scaffolds [36].

Tracheae for decellularisation were obtained from terminated New Zealand White (NZW) rabbits (Envigo, Huntingdon, UK). Tracheae were stripped of all fascia and washed three times in PBS containing penicillin and streptomycin (1X, Gibco) over 24 h. Tracheae were then decellularised using a two-cycle enzymatic detergent decellularisation protocol previously published by Conconi *et al*. [37]. Briefly, tracheae were placed in a 4% sodium deoxycholate solution (S1827, Sigma-Aldrich) for 4 h with luminal agitation at room temperature, washed in PBS for 30 min with luminal agitation at room temperature, placed in a solution of 1 L of 1M sodium chloride containing 22.5 mg deoxyribonuclease I (9 003 989, Sigma) for 3 h and then washed in Milli-Q water overnight at 40 °C before repeating the above steps for the second cycle. After completing the second cycle, the tracheae were washed in Milli-Q water for 3 days at 40 °C with the water being changed daily. Finally, they were sterilised using ionising radiation.

### Assembly of tissue-engineered respiratory mucosa

Decellularised dermis prepared using the method described above was clipped into CellCrowns™ (Sigma-Aldrich) fitting either 24-well plates for *in vitro* experiments or 12-well plates for *in vivo* experiments. HBECs and lung fibroblasts were co-seeded onto the dermis at densities of 1×10^6^ per cm^2^ and 1×10^4^ per cm^2^, respectively, in 1 mL of epithelial cell culture medium. Scaffolds were maintained submerged in epithelial cell culture medium containing 5 µM Y-27632 [23] and the medium was changed daily. After 7 days, the CellCrowns™ were removed from the plates and those containing the scaffolds were placed into 0.4 µm PET membrane transwell inserts (Corning) for ALI culture [38]. Medium was changed to ALI medium, which was changed three times per week. Beyond 2 weeks at ALI, scaffolds were cleared of mucus by gentle aspiration every other day. After 3–4 weeks at ALI, high-speed videos were taken using an inverted microscope system (Nikon Ti-U 1000, Nikon, Japan) with a 40× objective and a 1.5× magnifier to assess ciliary beat frequency and pattern. The digital camera (Motion Pro 4x; IDT, Pasadena, USA) was set for 512×512 regions of interest and recorded at 250 frames·s^–1^ for ciliary beat frequency and 500 frames·s^–1^ for ciliary beat pattern. Ciliary beat frequency was analysed using CiliaFA [39] and also calculated by determining the number of frames required for five full sweeps of the ciliary tip. Ciliary dyskinesia was assessed by defining the uncoordinated ciliary beat pattern across a strip of ciliated epithelium. The dyskinesia score was calculated from the percentage of dyskinetic ciliated cells against the total number of ciliated cells.

### Histology

Samples for histology were washed twice in PBS and fixed in 10% neutral buffered formalin (NBF) for 1 h. Epithelialised scaffolds were fixed for 30 min and embedded in HistoGel (ThermoFisher Scientific) to protect the top layer of epithelium from detachment during processing. After fixation, samples were stored in 70% ethanol at 4 °C before dehydration using an automated system (Leica TP 1050, Leica, London, UK). Formalin-fixed, paraffin-embedded samples were sectioned at 5 µm thickness. Haematoxylin and eosin staining was performed using an automated Sakura DRS-601 staining machine (Sakura, Alphen aan den Rijn, The Netherlands). Samples were mounted using a Sakura Coveraid (Sakura) automatic cover-slipping machine with Tissue-Tek cover slip film (Bayer Diagnostics, Reading, UK).

### Immunofluorescence

Slides were dewaxed for immunofluorescence using an automated process (DRS-601, Sakura) and then washed in distilled water and blocked with PBS containing 10% FBS (block solution) for 2 h. Primary antibodies were applied in block solution and incubated at 4 °C overnight. Following three PBS washes, species-specific secondary antibodies (AlexaFluor, ThermoFisher Scientific) were applied in block solution at a concentration of 1:200 for 1 h at room temperature. Slides were washed a further two times in PBS and stained with 4′,6-diamidino-2-phenylindole (DAPI) (5 mg·mL^−1^ stock, 1:10 000 in PBS; Life Technologies) for 10 min. Following a final PBS washing step, slides were cover-slipped using ImmuMount (GeneTex, Irvine, USA), sealed with nail varnish and imaged on a Zeiss 700 confocal microscope (Zeiss, Oberkochen, Germany).

### Immunocytochemistry

For top-down images, scaffolds were washed twice in PBS, fixed in 10% NBF for 30 min, washed twice in PBS for 5 min each and incubated in block solution for 2 h at room temperature. Primary antibodies were applied in block solution and incubated overnight at 4 °C. After three 5-minute washes in PBS, species-specific secondary antibodies were applied at 1:200 in block solution at room temperature for 1 h. Scaffolds were washed twice in PBS and DAPI (5 mg·mL^−1^ stock, 1:10 000 in PBS; Life Technologies) was applied. Samples were washed in PBS before imaging, either *in situ* or following removal from wells and cover-slipping using ImmuMount (GeneTex, Irvine, USA), using a Zeiss 700 confocal microscope (Zeiss).

To assess cell viability on the surface of scaffolds, a live/dead cell viability assay was used (ThermoFisher Scientific). Calcein AM (3.4 µL·mL^−1^) and ethidium homodimer-1 (4 µL·mL^−1^) were mixed with medium and added to scaffolds following cell adhesion. The scaffolds were incubated for 45 min and mounted on glass slides for top-down, whole-mount imaging on a Zeiss 700 confocal microscope (Zeiss).

### Scanning electron microscopy

Scaffolds were fixed in 2.5% glutaraldehyde, washed with 0.1 M phosphate buffer (pH 7.4) and distilled water, dehydrated in a graded ethanol–water series to 100% ethanol and dried using carbon dioxide. Using sticky carbon tabs, the scaffolds were then mounted on aluminium stubs and coated with a 2 nm layer of gold/palladium using an ion beam coater (Gatan, California, USA). The scaffold surface was imaged using a Jeol 7401 field emission scanning electron microscope (Jeol, Welwyn Garden City, UK). Ciliary length measurements were made at 20 000× screen magnification by electron microscopy.

### Chick chorioallantoic membrane assay

Fertilised Bovan Brown chicken eggs were supplied by Henry Stewart & Co (Norfolk, UK). In brief, at Embryonic Day 3, eggs were cleaned with 70% ethanol and 3–4 mL of albumin was removed *via* needle aspiration at the apex of the shell. A 2×2 cm window was excised from the upper aspect of the shell to reveal the embryo and chorioallantoic membrane (CAM) blood vessels. Windows were sealed with adhesive tape and the eggs were incubated for a further 5 days. At Embryonic Day 8 the CAM was re-exposed and minutia pins were used to disrupt several of the smaller blood vessels. Sections of test scaffold (2 mm^2^) were placed gently onto the CAM with the epithelial side upwards and excess blood was drained. The window was re-sealed and the eggs incubated until Embryonic Day 10. Following imaging with a Leica Zoom 2000 stereomicroscope (Leica) with camera adaptor (Magnifi™, Arcturus Labs, Kansas, USA), grafted CAMs were harvested and fixed overnight in 4% paraformaldehyde (PFA). A section of sterile plastic casing was used as a negative control and acellular decellularised dermis was used to examine the effect of including human cells. Comparison of epithelialised scaffolds was performed in two separate experiments involving at least six eggs in each experimental group. Comparison of acellular dermis was performed in one experiment with at least six eggs in each experimental group.

### Engraftment of tissue-engineered respiratory mucosa on decellularised trachea in rabbits

Male NZW rabbits aged 9–10 weeks weighed 2.5–3 kg on arrival. General anaesthesia was induced using 0.5 mL·kg^−1^ ketamine intramuscularly and 0.2 mL·kg^−1^ of xylazine, and was maintained with sevoflurane (all drugs supplied by National Veterinary Services, Stoke-on-Trent, UK). Sections of decellularised trachea (5 cm) were pre-implanted in a lateral thoracic muscle flap raised from the chest and tunnelled under the cervical skin into the neck in order to create a vascularised scaffold on which to graft engineered mucosa. A Foley catheter (size 16F) was inserted through the lumen, cut to size and sutured in place at either end with 4.0 Prolene sutures (Ethicon, Berkshire, UK) to act as a stent and prevent scaffold collapse. The trachea was then wrapped in the muscle flap and secured within the neck using a 3.0 silk suture (Ethicon) and the wound closed. Concurrently, HBECs and fibroblasts were seeded onto decellularised dermis to fabricate the respiratory mucosal grafts as described above. After 4 weeks of pre-vascularisation, the cervical incision was re-opened and the scaffold identified in the muscle wrap. The trachealis was identified and opened by incision directly through muscle. The tracheal rings were visually assessed for evidence of vascularisation, structural integrity and signs of infection, before the newly fabricated tissue-engineered mucosa was grafted onto the luminal surface. A Foley catheter stent or Kaltostat alginate-based dressing (Convatec, Flintshire, UK) was placed over the graft to protect the epithelial layer and a stent placed to prevent displacement of the graft within the muscle flap. The tracheal and cervical incisions were closed and the animals were terminated either 24 h or 1 week post-transplantation.

Immunosuppression of rabbits was required to reduce the probability of acute rejection of the transplanted human cells [40]. Tacrolimus (Cambridge Bioscience, Cambridge, UK) was administered subcutaneously at 0.3 mg·kg^−1^ for three consecutive days, beginning 7 days before the day of surgery and then on alternate days until the end of the experiment. Dexamethasone was co-administered in later experiments at a dose of 2 mg·kg^−1^ three times daily, beginning 24 h before grafting (supplementary table S2). Blood samples were taken on the day of graft implantation and sent to the Royal Veterinary College (Hatfield, UK) for quantification of white cells, lymphocytes and neutrophils.

### Engraftment of tissue-engineered respiratory mucosa in immunosuppressed mice

NOD SCID mice (Envigo) were anaesthetised and maintained with sevoflurane (National Veterinary Services). A 5-cm transverse incision was made on the back of the mouse and a subcutaneous pocket made. Decellularised dermis-based respiratory mucosal sheets were then laid onto the surface of the muscle and a layer of Mepitel silicone dressing (Mölnlycke Healthcare, Gothenburg, Sweden) was laid over the top to prevent shear. After 7 days, the mice were terminated, the wound was reopened and the graft and underlying mouse muscle were explanted for histological processing.

### Statistics

Statistical analyses were performed using GraphPad Prism (GraphPad Software, San Diego CA, USA) as indicated in the figure legends. p-Values are reported rounded to three decimal places and statistical significance was assigned for p<0.05. All *in vitro* experiments were carried out in at least technical triplicates unless otherwise indicated and repeated as described in the figure legends. Data are presented as mean±sem values.

## Results

### Extracellular matrix dependent human airway epithelial cell attachment, proliferation and differentiation

**FIGURE 1:**
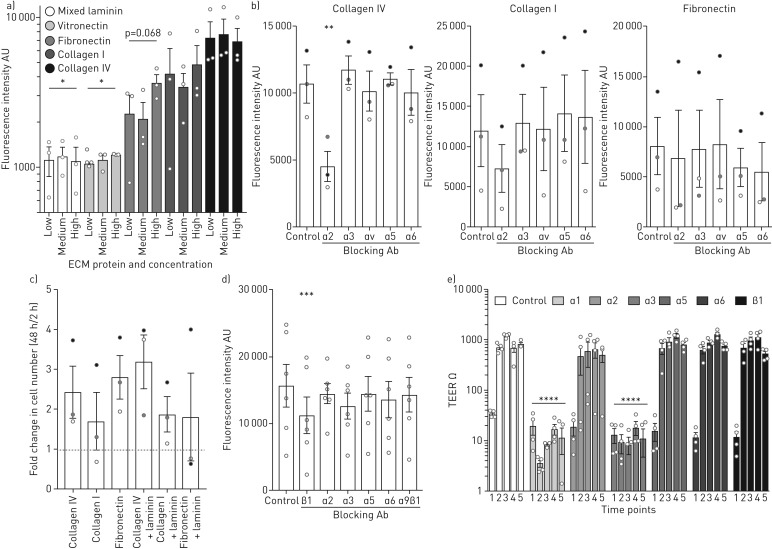
The role of extracellular matrix (ECM) proteins in human bronchial epithelial cell (HBEC) behaviour. a) HBECs (5×10^4^) were seeded on non-adherent tissue-culture plastic coated with ECM proteins and attachment was determined at 30 min using CYQUANT GR (ThermoFisher Scientific, Hemel Hempstead, UK) with background fluorescence subtracted. Attachment to collagen IV was greater than to laminin (two-way ANOVA with Holm-Sidak's test for multiple comparisons) and vitronectin-coated wells. The experiment was repeated three times in technical triplicate, including using two independent donor cell cultures. Data are presented on a log scale. b) HBECs were incubated in blocking antibodies against integrins α2, α3, αv, α5 and α6 for 20 min before cells (2×10^4^) were seeded onto non-adherent tissue-culture plastic wells coated with either collagen IV, collagen I or fibronectin. Fluorescence with background subtracted was measured using CYQUANT GR. Blocking integrin α2 led to significantly less HBEC attachment in the collagen IV-coated wells (one-way ANOVA with Holm-Sidak's test for multiple comparisons), but not the collagen I- (p=0.141) or fibronectin-coated wells (p=0.881), compared to control wells. The experiment was repeated three times in technical triplicate (indicated in black, grey and white), including using two independent donor cell cultures. c) HBEC cell number was determined using CYQUANT GR and a standard curve of known cell number at 2 h and 48 h after seeding HBECs (1×10^4^) onto non-adherent tissue-culture plastic coated with ECM proteins. No statistical difference was found between the groups (one-way ANOVA with Holm-Sidak's test for multiple comparisons; p>0.25 for all comparisons). The experiment was repeated three times in technical triplicate, including using two independent donor cell cultures. d) Blocking antibodies were added to wells 2 h after seeding HBECs (5×10^3^) onto collagen IV- and laminin-coated wells. Fluorescence with background subtracted was recorded at 48 h using CYQUANT GR and compared to a control well without blocking antibody. Significantly less epithelial cell proliferation was observed compared to the control in the presence of integrin β1-blocking antibody (one-way ANOVA with Holm-Sidak's test for multiple comparisons). The experiment was repeated six times in technical triplicate, including using two independent donor cell cultures. e) Integrin blocking antibodies were added to air–liquid interface (ALI) cultures at the point of ALI creation and at every feed thereafter. Trans-epithelial resistance (TEER) as a measure of epithelial integrity was recorded at five timepoints after air-lift (1=0 days, 2=7 days, 3=14 days, 4=21 days and 5=28 days). Blockade of α1 and α3 integrins inhibited trans-epithelial resistance (two-way ANOVA with Holm-Sidak's test for multiple comparisons). The experiment was performed by making three repeated measurements from duplicate wells from two independent donor cell cultures (*i.e.* four independent wells at each timepoint). Data are presented on a log scale. *: p=0.018; **: p=0.002; ***: p=0.006; ****: p<0.001.

We first assessed the attachment of cultured primary human bronchial epithelial cells (HBECs) [41–43] to ECM proteins found in the native human airway (supplementary figure S1), finding that collagen IV-coated wells performed best with significantly more cells attaching than to laminin-coated or vitronectin-coated wells (figure 1a). To determine the integrin mediators of this ECM attachment, we performed attachment assays to ECM proteins in the presence of blocking antibodies against various integrin α subunits. Blocking integrin α2 led to significantly lower levels of epithelial cell attachment to collagen IV but blocking integrin subunits α3, αv, α5 and α6 had no effect (figure 1b). Other than a trend towards decreased attachment to collagen I in the presence of an integrin α2-blocking antibody, attachment to collagen I or fibronectin was variable between experimental replicates but unaffected by the presence of these blocking antibodies (figure 1b). To determine the effect of ECM proteins on early epithelial proliferation after attachment, HBECs were seeded onto wells coated with matrix proteins that supported epithelial cell attachment and cell number was determined after 2 h and 48 h. No significant change in HBEC number was seen when these proteins were combined with laminin to more closely mimic the native ECM (figure 1c) but blocking antibodies against integrin β1 significantly curtailed early HBEC expansion on collagen IV-coated wells and laminin-coated wells (figure 1d). No inhibition of proliferation was seen in the presence of blocking antibodies against integrins α2, α3, α5, α6 or α9β1 (figure 1d).

To determine the effect of integrin blockade on HBEC differentiation, blocking antibodies against integrins α1, α2, α3, α5, α6 or β1 were added to ALI cultures at the point of air exposure (and at medium changes thereafter). Epithelial integrity increases during the establishment of a polarised, differentiated epithelial layer through the formation of tight junctions. To examine this process, TEER was measured on Days 0, 7, 14, 21 and 28 after air exposure. Epithelial integrity was disrupted by inhibition of integrin α1 and integrin α3, both of which are expressed in native human airway epithelium (supplementary figure S2), indicating a failure of cells to differentiate appropriately. TEER values were unaffected in the presence of blocking antibodies against integrins α2, α5, α6 or β1 (figure 1e) and epithelial differentiation to towards multiciliated and mucosecretory cells was not obviously altered by these blocking antibodies, as evidenced by high-speed video microscopy at Day 21 and immunofluorescence staining of independent cultures incubated with blocking antibodies (supplementary figure S3). In wells treated with integrin α1- and α3-blocking antibodies, no evidence of ciliation was detected and cell adherence to the PET membrane had largely failed consistent with TEER readings (figure 1e).

### Extracellular matrix proteins and attachment in tissue-engineered scaffolds

**FIGURE 2:**
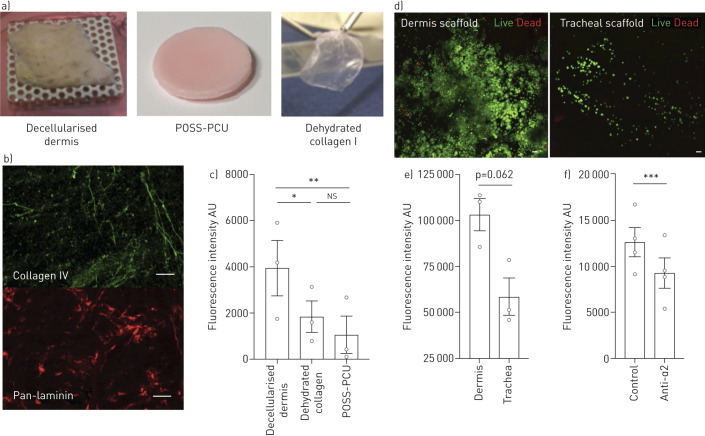
Attachment of human bronchial epithelial cells (HBECs) to tissue-engineered scaffolds. a) Digital photographs of the three scaffolds tested: decellularised dermis (biological), POSS-PCU (synthetic) and dehydrated collagen I (biomimetic). b) Whole-mount, top-down immunofluorescence staining of the basement membrane proteins collagen IV and pan-laminin in decellularised dermis indicates that these matrix proteins are preserved following decellularisation. c) HBECs (5×10^4^) were seeded into each scaffold-containing well. After 1 h, the wells were washed and trypsin was applied to detach the HBECs that had adhered to the scaffolds. In a separate 96-well plate, CYQUANT GR (ThermoFisher Scientific, Hemel Hempstead, UK) was used to measure fluorescence of attached cells with values from unseeded control scaffolds subtracted. Fluorescence was significantly greater in the decellularised dermis group compared with the collagen I (one-way ANOVA with Holm-Sidak's test for multiple comparisons) and POSS-PCU scaffolds. The experiment was repeated three times in technical triplicate, including using two independent donor cell cultures. d) Whole-mount, top-down confocal images showing live/dead staining of decellularised dermis and tracheal scaffolds at 2 h post-epithelial cell seeding. e) Cell viability of epithelial cells (1.5×10^5^) seeded on decellularised dermis or trachea was compared using an alamarBlue (ThermoFisher Scientific) cell viability assay. Fluorescence intensity was measured at 2 h following adherence and values from negative control scaffolds were subtracted. Fluorescence intensity was greater in the dermis group compared to the tracheal group but the finding did not reach statistical significance (two-tailed, paired t-test). The experiment was repeated three times in technical triplicate, including using two independent donor cell cultures. f) HBECs (1.5×10^5^) were seeded onto decellularised dermis scaffolds with and without pre-treatment with an integrin α2-blocking antibody. Cells were washed at 1 h and an alamarBlue cell viability assay was performed with background fluorescence subtracted. Blocking integrin α2 resulted in significantly less attachment of cells to decellularised dermis (paired t-test). The experiment was performed four times in technical triplicate, including using two independent donor cell cultures. All scale bars=50 µm. *: p=0.029; **: p=0.015; ***: p=0.006.

To move our experiments towards airway mucosal regeneration, we investigated attachment of epithelial cells to three relevant scaffolds of varying ECM complexity (figure 2a): a complex biological scaffold that retained multiple ECM proteins including collagen IV and laminin after decellularisation (decellularised dermis) (figure 2b); a simpler collagen I matrix; and a synthetic POSS-PCU scaffold that did not contain ECM proteins. Epithelial cell attachment to the complex decellularised scaffold was greater than to the collagen I and POSS-PCU scaffolds (figure 2c), suggesting that the functionality of the ECM is retained despite possible alterations in its structure caused by decellularisation. We further investigated how decellularised dermis performed compared to decellularised trachea. After live/dead staining, abundant live cells were seen on the surface of decellularised dermis scaffolds, whilst only sparse coverage was seen on the decellularised tracheal scaffold (figure 2d). Correspondingly, in alamarBlue cell viability assays, there was a trend towards higher fluorescence intensity at 2 h following cell seeding onto decellularised dermis scaffolds compared to decellularised tracheal scaffolds (figure 2e). This indicated better adherence and/or cell survival on decellularised dermis, which was consistent with the two decellularisation protocols preserving matrix to different extents (supplementary figure S4). Similar to experiments on collagen IV (figure 1b), epithelial cell attachment to decellularised dermis was lower following blockade of integrin α2 (figure 2f).

### Culture of tissue-engineered respiratory mucosa

**FIGURE 3:**
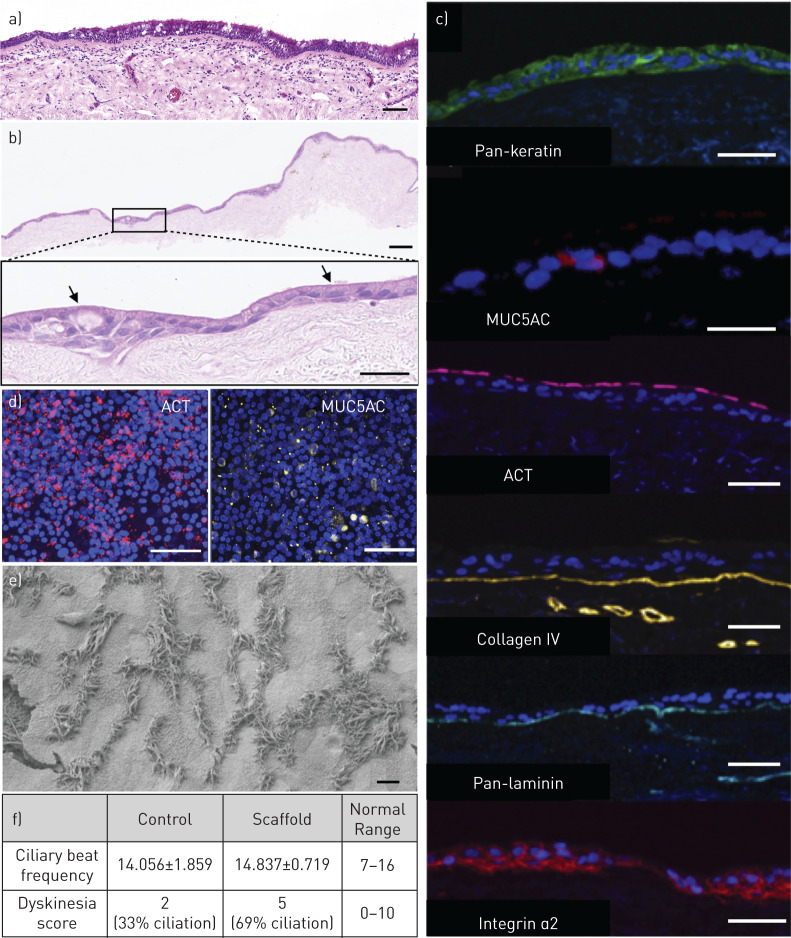
Human bronchial epithelial cell (HBEC) differentiation on decellularised dermis scaffolds at the air–liquid interface (ALI). a) Haematoxylin and eosin stained section of adult human tracheal mucosa. A pseudostratified layer of ciliated epithelium with mucus-secreting goblet cells can be seen overlying the basement membrane. The lamina propria lies underneath the basement membrane. b) Haematoxylin and eosin stained section of the respiratory mucosa cultured on decellularised dermis. A differentiated epithelial layer is seen along the length of the scaffold and cells with the morphological appearance of mucus-secreting goblet cells and ciliated cells are seen. c) Immunofluorescence staining of sections of bioengineered respiratory mucosa. The 4′,6-diamidino-2-phenylindole (DAPI)-positive cell layer stained positively for the epithelial marker pan-keratin (green), the mucosecretory cell associated mucin 5AC (MUC5AC) (red), the ciliary protein acetylated tubulin (ACT) (red), the basement membrane proteins collagen IV (yellow), pan-laminin (blue) and integrin α2 (red). d) Top-down confocal microscopy for markers of differentiation on decellularised dermis-based respiratory mucosa demonstrated ACT (red) and MUC5AC (yellow) positivity. e) Electron microscopy of decellularised dermis-based respiratory mucosa. Cilia can be seen on the surface of the scaffold. f) High-speed digital video analysis of one donor HBEC culture differentiated on either standard positron emission tomography (PET) ALI substrates (controls; n=5 videos) or decellularised dermis scaffolds (n=10 videos). Analysis of ciliary beat frequency and dyskinesia demonstrated that on decellularised dermis cilia exhibited a comparable beat frequency to the control substrate. The dyskinesia score was slightly higher than in controls but remained within normal range. Scale bars=50 µm in (a)–(d) and 10 µm in (e).

Based on these findings, we investigated the use of decellularised dermis as a substrate for respiratory mucosal regeneration. HBECs and primary human lung fibroblasts [44, 45] were cultured on decellularised dermis at an ALI for 3 weeks in order to induce differentiation of basal cells to mature epithelium containing mucosecretory and ciliated cells [38], as is seen in the native human airway (figure 3a). Consistent with previous observations using this scaffold material [46], mucus was observed on the surface of the scaffold after 2 weeks and by 3 weeks ciliary beat could be observed by top-down, high-speed digital video (supplementary material). Haemotoxylin and eosin staining of sections taken 3 weeks post-ALI demonstrated an intact epithelial layer across the extent of the scaffold, with both ciliated cells and mucus-containing secretory cells (figure 3b). The fate of fibroblasts remained uncertain however, as although fibroblasts had not convincingly repopulated the lamina propria (figure 3b), very rare cells with a fibroblastic morphology were observed by immunofluorescence (supplementary figure S5). The height of the pseudostratified layer was variable along the length of the scaffold but immunofluorescence staining demonstrated the expression of keratin, mucin 5AC (MUC5AC), indicating the presence of mucosecretory cells, acetylated tubulin (ACT) in cilia and the basement membrane ECM proteins collagen IV and laminin (figure 3c). Expression of integrin α2, identified earlier as an important mediator of epithelial attachment (figure 1b) was also seen throughout the differentiated epithelium (figure 3c). Top-down immunofluorescence confirmed the presence of ACT-positive ciliated cells and MUC5AC-positive mucosecretory cells across the surface of the scaffold (figure 3d). Electron microscopy of the mucosal sheets further confirmed the presence of ciliated cells on the apical surface of the dermis-based scaffold (figure 3e). Ciliary length measurements were made on between seven and 10 cilia per cell from six different ciliated cells from five separate strips of epithelium. Cilia from three of the cells were of normal length (length±standard deviation (sd): 5.5±0.3 µm, 5.6±0.3 µm, 5.7±0.4 µm) and cilia from three cells were slightly shorter (5.4±0.3 µm, 5.0±0.2 µm, 5.0±0.3 µm) suggesting ciliogenesis might be on-going or incomplete in some cells. Importantly, analysis of ciliary beat frequency showed that it was comparable to that seen using a standard PET ALI culture substrate (figure 3f). Finally, the ciliary dyskinesia score of cells on decellularised dermis scaffolds was within the anticipated range for ciliated ALI cultures (figure 3f).

### Grafting of tissue-engineered respiratory mucosa onto chick chorioallantoic membrane

**FIGURE 4:**
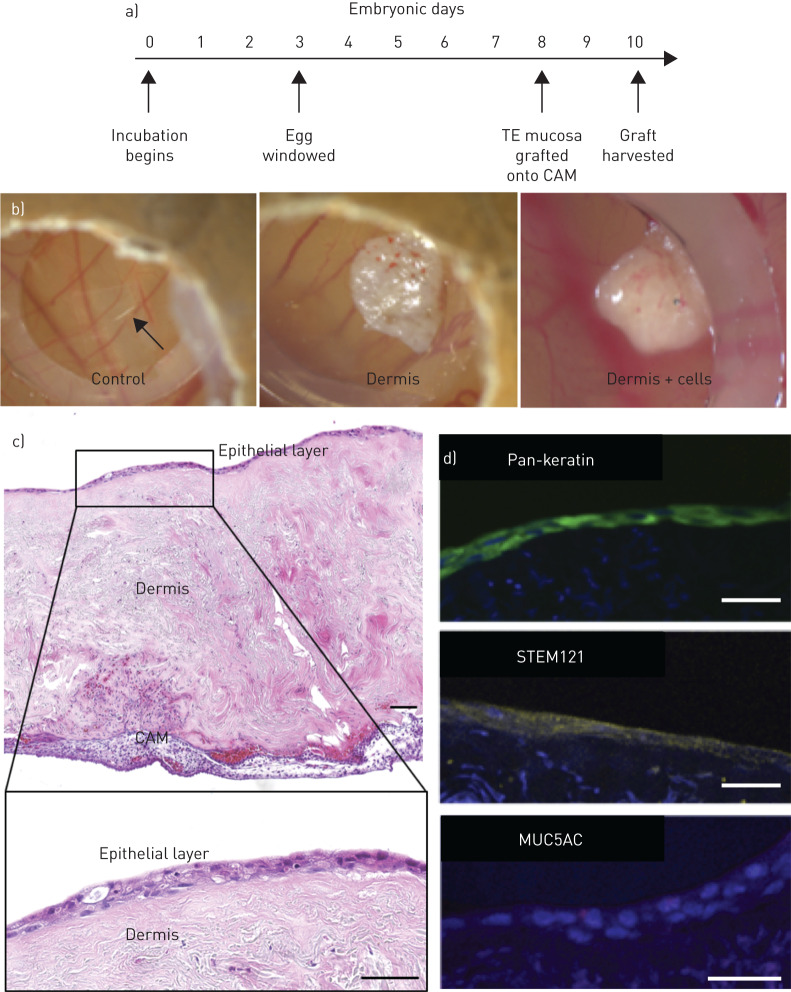
Engraftment of decellularised dermis-based airway mucosal grafts in the chick chorioallantoic membrane assay (CAM). a) Timeline of the chick CAM assay. The egg is incubated and then windowed at Embryonic Day 3, while the scaffold is grafted at Embryonic Day 8 before harvest at Embryonic Day 10. b) Digital photographs of CAM assays after 2 days. c) Haematoxylin and eosin stained sections of the respiratory mucosal layer grafted onto CAM assay at Embryonic Day 10. An epithelial layer is demonstrated on the surface of the dermis overlying the CAM. Higher magnification of the epithelial layer demonstrates preservation of an epithelium but apparent loss of cilia. d) Immunofluorescence staining of sections of decellularised dermis-based respiratory mucosa. The 4′,6-diamidino-2-phenylindole (DAPI)-positive cell layer stained positively for the epithelial marker pan-keratin (green), the human cell marker STEM121 (yellow) and rarely the mucosecretory cell marker mucin 5AC (MUC5AC) (red). The CAM experiment involving epithelialised scaffolds was performed twice including six eggs in each experimental group on each occasion. The decellularised dermis CAM experiment was performed once with six eggs in each experimental group. All scale bars=50 µm. TE: tissue-engineered.

Having determined that the decellularised dermis matrix was capable of supporting a differentiated respiratory epithelium *in vitro*, we next assessed short-term engraftment of decellularised dermis scaffolds seeded with HBECs and lung fibroblasts onto a vascularised surface in a chick CAM assay (figure 4a). The CAM assay has the advantages of immune naivety, rapid neo-vascularisation and exposure of engrafted tissue to an air interface. At 48 h post-graft, top-down digital photography showed small blood vessels penetrating the scaffold, indicating neo-vascularisation (figure 4b), in contrast to plastic negative controls that showed no such vascularisation (figure 4b). Sections of acellular decellularised dermis also demonstrated neo-vascularisation (figure 4b), indicating that this process was not dependent on the inclusion of transplanted cells. Haematoxylin and eosin stained sections of the engineered respiratory mucosa at this time-point demonstrated preservation of the epithelium (figure 4c). Furthermore, immunofluorescence staining confirmed their human (STEM121) (figure 4d) and epithelial (pan-keratin) (figure 4d) origin and showed rare cells expressing the mucosecretory cell-associated protein MUC5AC (figure 4d). However, an absence of ACT^+^ cilia suggested that epithelial remodelling had occurred in the *in vivo* environment.

### Grafting of tissue-engineered respiratory mucosa *in vivo*

**FIGURE 5:**
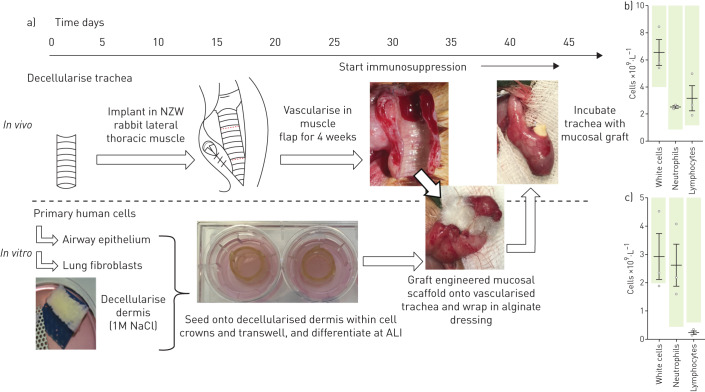
Timeline for engrafting decellularised dermis-based airway mucosal grafts onto a pre-vascularised segment of decellularised trachea in rabbits. a) Cadaveric rabbit trachea was decellularised using a series of enzymatic and detergent washes. Decellularised tracheae were pre-implanted in the lateral thoracic muscle of New Zealand White (NZW) rabbits to promote vascularisation. At this time, human bronchial epithelial cells (HBECs) and lung fibroblasts were seeded onto decellularised dermis and cultured at an air–liquid interface (ALI). After 4 weeks, the muscle and tracheal composite was opened and the trachea visually assessed for signs of vascularisation, infection and for structural integrity ahead of grafting the cultured respiratory mucosal graft onto the luminal surface. In some rabbits, alginate dressing was placed over the mucosal scaffold to protect the epithelial cells and then stents were placed within the lumen to secure the graft in place. Immunosuppression began prior to transplantation of bioengineered mucosal grafts. b) Immunosuppression efficacy with tacrolimus in rabbits (n=3; 0.3 mg·kg^−1^ subcutaneously for 3 days and then on alternate days; blood sampled on Day 7). Green bars indicate normal range. c) Immunosuppression efficacy with combined tacrolimus and dexamethasone in the rabbit model (n=3; 0.3 mg·kg^−1^ tacrolimus subcutaneously for 3 days and then on alternate days; three doses of 2 mg·kg^−1^ dexamethasone intramuscularly 24 h prior to blood sampling on Day 7). Green bars indicate normal range.

**FIGURE 6:**
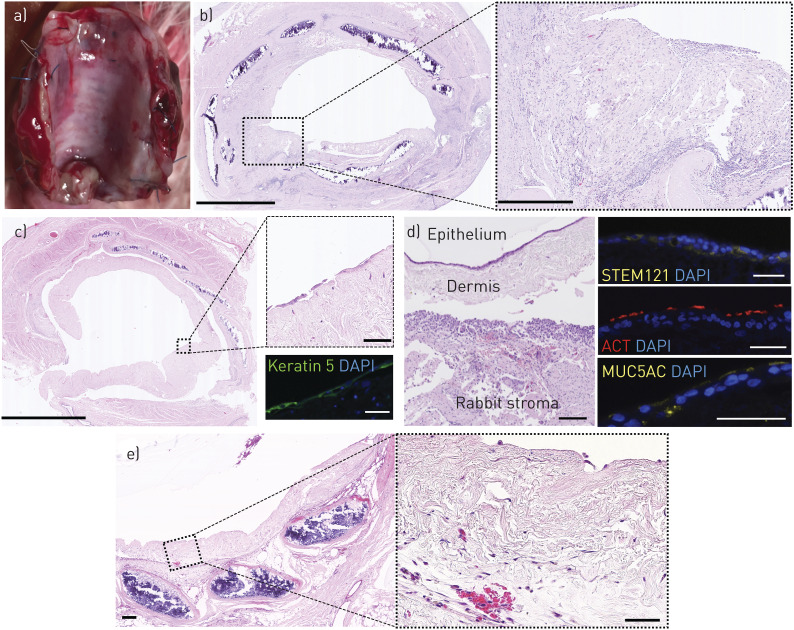
Engraftment of decellularised dermis-based airway mucosal grafts onto pre-vascularised decellularised tracheae in rabbits. a) Digital photograph of an explanted trachea 7 days after grafting with bioengineered airway mucosa. The grafted scaffold can be seen on the luminal surface as a distinct layer overlying the cartilage rings. b) Haematoxylin and eosin staining of explanted rabbit trachea 7 days after grafting tissue-engineered respiratory mucosa (TERM) around a silicone stent with the use of tacrolimus immunosuppression. The grafted TERM can be seen on the luminal surface with an intense inflammatory infiltrate and loss of all epithelium (magnified section). Scale bars=1 mm (left) and 250 µm (right). c) Rabbit trachea 7 days after grafting TERM around a silicone stent with the use of tacrolimus and dexamethasone immunosuppression. The grafted TERM can be seen on the luminal surface with less inflammatory infiltrate and some overlying epithelium that was incomplete over large portions (magnified area). Scale bars=1 mm (left) and 100 µm (upper right). Sections were stained for keratin 5 (green) and 4′,6-diamidino-2-phenylindole (DAPI) (blue). Keratin 5+ cells were found over small areas of the trachea (green). Scale bar=50 µm (lower right). d) Haematoxylin and eosin staining of an explanted rabbit trachea 24 h after grafting with an alginate dressing and dual immunosuppression. An epithelial layer was observed on the surface of one of the grafts but the grafts had detached from the underlying trachea, perhaps during processing. Immunofluorescence staining for the human cell marker STEM121 (yellow), mucin 5AC (MUC5AC) (yellow) and the ciliary protein acetylated tubulin (ACT) (red) is shown. Scale bars=50 µm. e) Haematoxylin and eosin staining of explanted trachea 5 days after grafting with an alginate dressing and dual immunosuppression. The epithelial layer had been lost from the decellularised dermis. The magnified image shows evidence of blood cells within the dermis layer and the presence of inflammatory cells. Scale bars=50 µm.

Finally, we investigated whether we could apply these decellularised dermis-based respiratory mucosal sheets to a pre-vascularised, decellularised trachea in an *in vivo* rabbit model [47] (figure 5 and supplementary table S2). If successful mucosal regeneration were achieved in this manner, the construct could be rotated on its vascular pedicle to replace a section of damaged trachea and provide a re-mucosalised and vascularised section of airway at the point of transplantation. Segments of decellularised rabbit trachea 5-cm in length were implanted into muscle flaps in the necks of NZW rabbits. We allowed 4 weeks for scaffold vascularisation and initiated immunosuppression before surgical grafting. To monitor immunosuppression, venous blood was sampled on the day of mucosal grafting. In our initial experiment, immunosuppression consisted of tacrolimus only and lymphocyte counts were within normal range (figure 5b). After 7 days, grafts on tracheae had integrated macroscopically (figure 6a) but did not show a preserved epithelial layer (two out of two) (figure 6b). An intense inflammatory reaction was observed and subsequently the immunosuppressive regime was modified to include dexamethasone. The combination of dexamethasone and tacrolimus caused lymphopenia (figure 5c) and led to a less intense inflammatory reaction within the implanted dermis layer at 7 days (figure 6c). Very sparse areas of keratin-positive epithelial cells could be found in these rabbits (two out of three) (figure 6c). To counteract the possibility of mechanical shear damaging the engrafted epithelial layer, we finally implanted decellularised dermis-based airway mucosal sheets with a protective layer of soft alginate dressing in place of a silicone stent. At 24 h after engraftment, the epithelial layer was preserved in one out of two rabbits (figure 6d) and demonstrated appropriate expression of markers associated with mucosecretory and ciliated differentiation (figure 6d). However, vascular channels did not appear to penetrate to the surface and the mucosal layer was detached from the underlying stromal layer following processing and sectioning, possibly indicating weak integration (figure 6d). After 5 days, the bioengineered graft was well-integrated with some evidence of re-vascularisation (figure 6e) but the epithelial layer had been lost (two out of two) (figure 6e). Blood cells and diffuse inflammatory cell infiltration were observed within the decellularised dermis at this time point (figure 6e).

**FIGURE 7:**
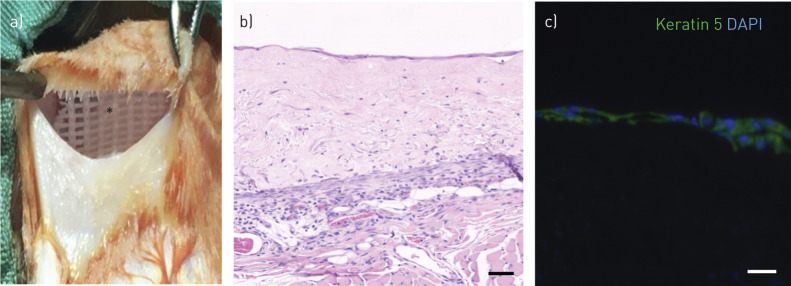
Engraftment of decellularised dermis-based airway mucosal grafts in NOD SCID mice. a) A 5-cm transverse incision was made on the back of three NOD SCID mice. A subcutaneous pocket was developed and the decellularised dermis scaffolds, pre-seeded with differentiated human bronchial epithelial cells (HBECs) and lung fibroblasts, were implanted onto vascularised muscle and overlaid with a silicone-based dressing (asterisk). b) Haematoxylin and eosin staining shows integrated scaffold after 7 days. A continuous monolayer epithelium was seen suggesting that differentiated cells may have been lost. c) Immunofluorescence staining confirmed that the monolayer was keratin 5-expressing (green). Slides were counterstained with 4′,6-diamidino-2-phenylindole (DAPI) (blue). All scale bars=50 µm.

Due to the possibility of incomplete immunosuppression leading to these results in rabbits, we further investigated *in vivo* integration in NOD SCID mice. Decellularised dermis-based airway mucosal sheets were grafted onto back muscle and overlaid with a silicone dressing to protect the epithelial layer (figure 7a). After 7 days, the grafts were retrieved and histology demonstrated retention of epithelial cells (three out of three) (figure 7b), although coverage of scaffolds varied among replicates. Retained epithelial cells expressed the basal cell-associated protein keratin 5 (figure 7c), suggesting that remodelling had occurred, perhaps because of the lack of polarity signals available to the epithelial cells in this experimental model.

## Discussion

Collagen IV and laminin anchor multiple epithelia, including airway, skin [21], small intestine [48] and cornea [49], to the underlying stroma *in vivo* [50]. Supporting their active role in epithelial remodelling, collagen IV expression is reduced in partial thickness dermal wounds as epithelial cells migrate [51] and laminin guides respiratory [52], epidermal [53] and corneal [49] epithelial cell migration. Our study suggests that collagen IV is a key mediator of airway epithelial cell attachment and proliferation on bioengineered scaffolds and that integrins α2 and β1 are mediators of these processes, consistent with the known role of integrin α2/β1 as a receptor for both ECM proteins [54].

We believe that the presence (and native presentation) of collagen IV and laminin in bioengineered airway constructs and/or manipulation of integrin α2 mediated adhesion, may improve epithelial cell attachment efficiency and permit lower cell seeding densities to achieve scaffold coverage [22]. The benefits of this are manifold as overall cell culture time, the number of population doublings experienced by cells in culture and manufacturing costs could be reduced. Although this could take many forms, we identified decellularised dermis as a promising scaffold material for airway regeneration based on its retention of collagen IV and laminin. Compared to collagen I-based scaffolds, synthetic scaffolds and decellularised tracheal scaffolds, primary HBECs adhered well to decellularised dermis *in vitro*. It is likely that the more extensive decellularisation process required for whole trachea disrupts the collagen IV-rich basement membrane, thus limiting epithelial adherence. Further research might aim to deploy decellularisation methods that preserve key matrix proteins on the luminal surface or to develop methods to replace these when they are lost through decellularisation.

Consistent with a previous report [46], we generated bioengineered respiratory mucosa with beating cilia and mucus secretion by culturing primary HBECs on decellularised dermis at an ALI *in vitro*. We found that the length of cilia was comparable to those in native airways and ciliary beat frequency and dyskinesia scores were comparable to healthy human nasal cilia as assessed by high-speed video microscopy either directly [55] or after culture at ALI [22]. Such scaffolds may be manipulated surgically and could be a means to restore mucociliary coverage in respiratory mucosa that has been lost or damaged. To this end, we examined the performance of the scaffolds in CAM assays, finding that the epithelium was retained 2 days post-engraftment but that cilia were no longer present. The fate of these cells is unknown but our data suggest that the CAM assay might act as an accessible short-term epithelial remodelling assay in the window before 15 days when immune reactions to implanted material become limiting [56].

In a second *in vivo* study, we used a two-stage transplantation procedure in rabbits to test a decellularised dermis-based bioengineered respiratory mucosal graft. The combination of tacrolimus and dexamethasone resulted in a lymphopenia with preserved neutrophil count that could limit immune-mediated remodelling of the scaffold whilst preventing immunosuppression-associated opportunistic infections. We saw engraftment with short-term epithelial retention but were unable to demonstrate longer-term retention of the differentiated epithelial layer. Delayed revascularisation may explain this observation, as penetration of new vascular channels approximately 1 mm into the dermis scaffold is likely to take several days, during which time the epithelium relies on diffusion of nutrients from the underlying graft bed [57]. Our team's experience in producing 3-D tissue-engineered skin and oral mucosa, including clinical engraftment, shows that grafts placed on poorly vascularised beds often fail to survive [58]. The inclusion of endothelial cells and angiogenic growth factors has been shown to shorten the time for neo-vascularisation in synthetic and biological scaffolds and may be a potential strategy to more rapidly restore a blood supply to the epithelial layer [59, 60]. An alternative strategy would be to differentiate respiratory epithelial cells on a thinner scaffold that enables the delivery of nutrients and oxygen to the epithelial layer from the underlying capillaries by diffusion. Diffusion is limited to a distance of approximately 150 µm from the capillary and therefore the scaffold would need to be of similar thickness [61–63]. Whether taking a 150 µm shave of dermis to include the collagen- and laminin-rich basement membrane and the underlying lamina propria would shorten the time taken for revascularisation and enhance epithelial survival is a clear target for future investigations.

The frequent loss of the epithelial layer in our *in vivo* experiments highlights the technical challenges of grafting a new mucosal layer into the airway. In order to prevent displacement of a mucosal graft, a stent is deployed that retains the graft on the graft bed whilst maintaining airway patency. However, the dynamic properties of the airway inevitably result in mechanical shear of the stent against the engrafted epithelial layer that is likely to result in further epithelial loss [64]. Future developments might adopt custom-made stents with a supportive soft outer layer containing a nutrient supply that could enable engraftment and maintain airway patency while simultaneously supporting and protecting the epithelium. Alternatively, developing pro-angiogenic adhesives might allow grafting of the tissue-engineered respiratory mucosa onto the trachea without the need for a stent.

### Conclusions

Tissue-engineered whole organ transplants in the airways, but also in the bladder and bowel, are currently limited by the inability to regenerate a functioning mucosal lining. Our findings indicated that the ECM proteins collagen IV and laminin are important for respiratory epithelial adherence and expansion *in vitro*, and informed the development of bioengineered scaffolds that contain a differentiated respiratory mucosal layer on a decellularised dermis scaffold. The delivery of these scaffolds is feasible *in vivo* but, while epithelium was retained in the short-term, further research is required to improve long-term *in vivo* survival. Our work emphasises the importance of cell delivery methods in airway cell therapy research and suggests the merit of pre-clinical studies that directly compare differentiated cell transplantation to basal cell-only methods (although the success of the former would restore tissue function rapidly, undifferentiated basal stem cells might be more robust for transplantation).

## Supplementary material

10.1183/13993003.01200-2019.Supp1 **Please note:** supplementary material is not edited by the Editorial Office, and is uploaded as it has been supplied by the author.
Supplementary material. erj-01200-2019.supplement
Supplementary video. Top-down, high-speed digital video after three weeks of air-liquid interface culture of the decellularised dermis-based bioengineered mucosa. Ciliary motility can be observed across the surface the scaffold. erj-01200-2019.video

## Shareable PDF

10.1183/13993003.01200-2019.Shareable1 This one-page PDF can be shared freely online.
Shareable PDF ERJ-01200-2019.Shareable

## Supplementary Material

ERJ-01200-2019.Shareable.pdf
