# Supplementary material for: Bioengineered airway epithelial grafts with mucociliary function based on collagen IV- and laminin-containing extracellular matrix scaffolds
Source: Eur Respir J. 2020 Jun 18;55(6):1901200. doi: 10.1183/13993003.01200-2019 (PMC7301290; doi:10.1183/13993003.01200-2019)
Supplement: Supplementary file 1 [file erj-01200-2019.supplement.pdf]

Data Supplement

Bioengineered airway epithelial grafts with mucociliary function based on collagen IV- and laminin-containing extracellular matrix scaffolds

Nick J.I. Hamilton[a,b*], Dani Do Hyang Lee[c], Kate H.C. Gowers[a], Colin R. Butler[a], Elizabeth F. Maughan[a], Benjamin Jevans[d], Jessica C. Orr[a], Conor J. McCann[d], Alan J. Burns[d], Sheila MacNeil[e], Martin A. Birchall[b], Christopher O'Callaghan[c], Robert E. Hynds[a] and Sam M. Janes[a*]

a) Lungs for Living Research Centre, UCL Respiratory, University College London, London, U.K.

b) UCL Ear Institute, The Royal National Throat Nose and Ear Hospital, London, U.K.

c) Respiratory, Critical Care and Anaesthesia, UCL Great Ormond Street Hospital Institute of Child Health, London, U.K.

d) Stem Cell and Regenerative Medicine, Birth Defects Research Centre, UCL Great Ormond Institute of Child Health, London, U.K.

e) Department of Materials and Science Engineering, The Kroto Research Institute, North Campus, University of Sheffield, Sheffield, U.K.

<u>*Corresponding Authors:</u>

Dr. Nick J.I. Hamilton (nick.hamilton@ucl.ac.uk)

UCL Ear Institute, University College London, 332 Grays Inn Road, London, WC1X 8EE, U.K.

Professor Sam Janes (s.janes@ucl.ac.uk)

Lungs for Living Research Centre, UCL Respiratory, Division of Medicine, University College London, 5 University Street, London, WC1E 6JF, U.K.

Donor ID	Donor Type	Donor Site	Culture Method
1	Human adult	Bronchus	BEGM
2	Human adult	Bronchus	BEGM
3	Human adult	Bronchus	BEGM
4	Human adult	Bronchus	BEGM
5	Human adult	Small airway	BEGM
6	Human adult	Trachea	BEGM
7	Human adult	Bronchus	BEGM
8	Human adult	Bronchus	BEGM
9	Human adult	Bronchus	BEGM
10	Human adult	Bronchus	3T3+Y
11	Human adult	Bronchus	3T3+Y
12	Human adult	Bronchus	3T3+Y
13	Human adult	Bronchus	3T3+Y
14	Human adult	Bronchus	3T3+Y

Supplementary Table S1: Human bronchial epithelial cell donors.

Respiratory epithelial cells were isolated from the upper airways of human adult patients. Some donor cultures were isolated and expanded in bronchial epithelial growth medium (BEGM), while others were expanded in 3T3-J2 mouse embryonic feeder cell co-culture in the presence of Y-27632, a Rho-associated protein kinase (ROCK) inhibitor. For experiments involving in vitro attachment and proliferation, cells grown in BEGM were used, while for experiments requiring differentiation towards a ciliated epithelium, basal cells cultured in 3T3+Y were used due to their superior differentiation at late passage.

ID	1st Operation	2nd operation	Termination following grafting	Stenting	Immunosuppressive drugs	Outcome
1	Left sided thoracic flap raised with decellularized trachea wrapped in muscle	Graft TERM	7 days	Silicone Stent	Tacrolimus	No epithelial layer retained. Intense inflammatory infiltrate into the dermis layer.
2	Left sided thoracic flap raised with decellularized trachea wrapped in muscle	Graft TERM	7 days	Silicone Stent	Tacrolimus	
3	Left sided thoracic flap raised with decellularized trachea wrapped in muscle	Graft TERM	7 days	Silicone Stent	Tacrolimus	Died before endpoint due to wound dehiscence
4	Left sided thoracic flap raised with decellularized trachea wrapped in muscle	Graft TERM	7 days	Silicone Stent	Tacrolimus & dexamethasone	Minimal retention of epithelial cells, less intense inflammatory infiltrate
5	Left sided thoracic flap raised with decellularized trachea wrapped in muscle	Graft TERM	7 days	Silicone Stent	Tacrolimus & dexamethasone	
6	Left sided thoracic flap raised with decellularized trachea wrapped in muscle	Graft TERM	7 days	Silicone Stent	Tacrolimus & dexamethasone	
7	Left sided thoracic flap raised with decellularized trachea wrapped in muscle	Graft TERM	24 hours	Alginate Dressing	Tacrolimus & dexamethasone	Poor integration into underlying trachea
8	Left sided thoracic flap raised with decellularized trachea wrapped in muscle	Graft TERM	24 hours	Alginate Dressing	Tacrolimus & dexamethasone	Retention of epithelial layer, poor integration into underlying trachea
9	Left sided thoracic flap raised with decellularized trachea wrapped in muscle	Graft TERM	5 days	Alginate Dressing	Tacrolimus & dexamethasone	Loss of epithelial layer but integration of dermis with underlying trachea and evidence of vascular in growth
10	Left sided thoracic flap raised with decellularized trachea wrapped in muscle	Graft TERM	5 days	Alginate Dressing	Tacrolimus & dexamethasone	

Supplementary Table S2: ***In vivo*** **rabbit protocols.**

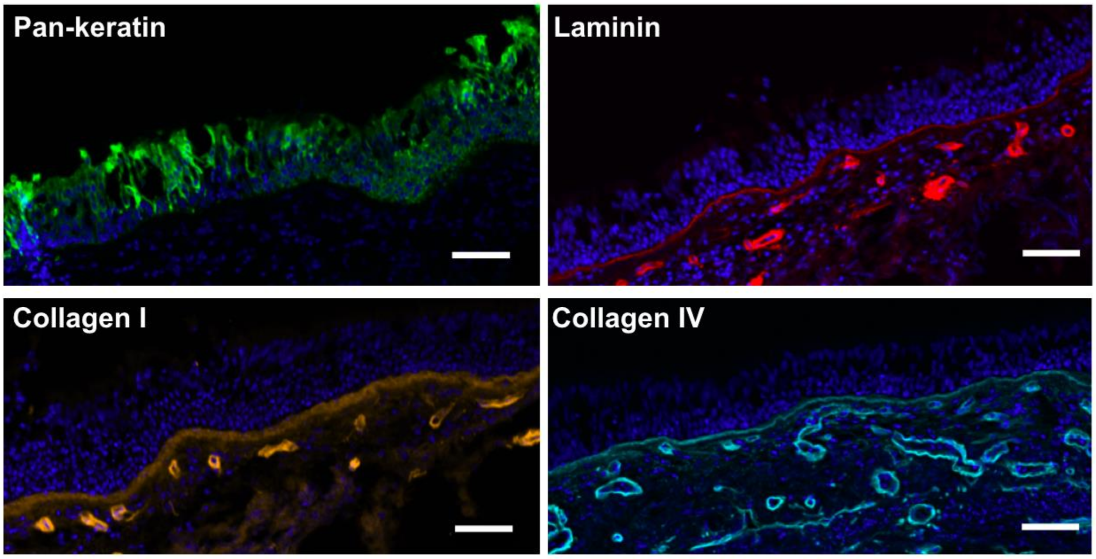

Supplementary Figure S1: ECM protein expression in the human tracheal mucosa.

Airway extracellular matrix (ECM) proteins were visualised in sections of formalin-fixed, paraffin-embedded human trachea. A pan-keratin antibody revealed epithelial cells (green; top left), while antibodies against the ECM proteins pan-laminin (red; top right), collagen I (orange; bottom left), and collagen IV (cyan; bottom right) established the localisation of these proteins in the basement membrane. Scale bars = 50 µm.

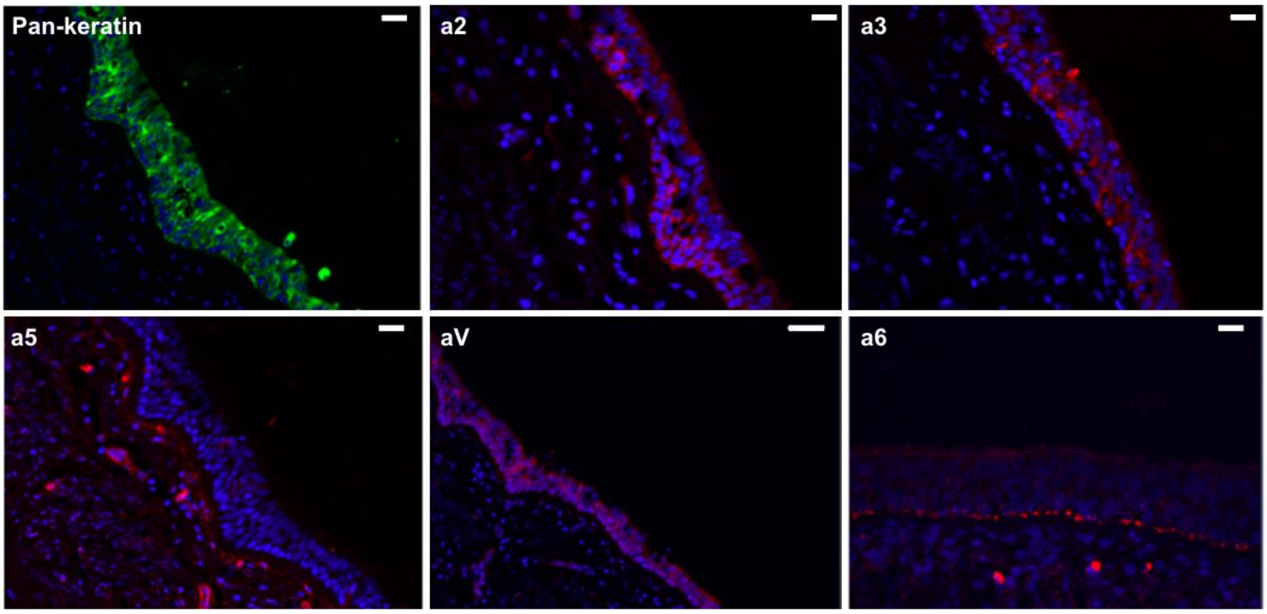

Supplementary Figure S2: Integrin subunit expression in human tracheal mucosa.

Expression of integrin subunits was assessed using immunofluorescence staining of sections of formalin-fixed, paraffin-embedded human trachea. A pan-keratin antibody revealed epithelial cells (green; top left), while antibodies against integrin α2 (red; top centre), α3 (red; top right), α5 (red; bottom left), αV (red; bottom centre) and α6 (red; bottom right) established the localisation of those proteins. Integrins α2, α3 and α6 were observed predominantly within the basal layer of the epithelium, while integrin αV was present throughout the epithelium. Integrin α5 was seen only within the lamina propria. Scale bars = 50 µm.

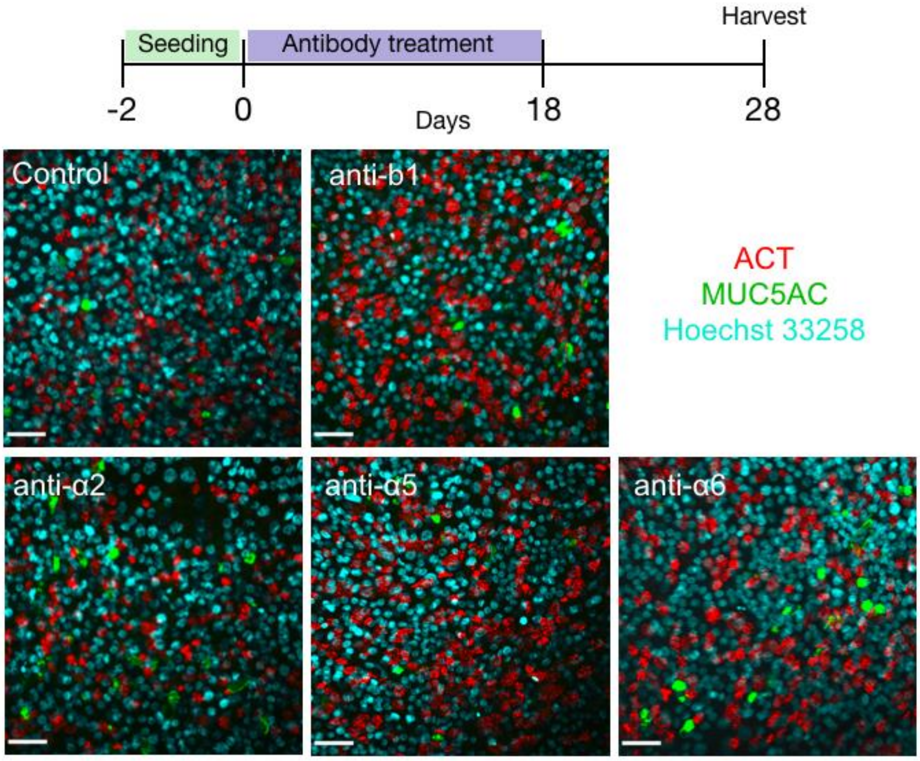

Supplementary Figure S3: Airway epithelial cell differentiation in ALI cultures incubated with blocking antibodies against integrin subunits α2, α5, α6 and β1.

ALI cultures were fixed and processed for immunofluorescence staining at Day 28, having been antibody-treated at each feed (three times per week) between Day 0 and Day 18. Cells were stained for acetylated tubulin (ACT; red), MUC5AC (green) and counterstained with Hoechst 33258 (nuclei; cyan). Blocking antibodies against integrins α1 or α3 were not tested here given the failure of epithelial cells treated with those antibodies to form intact epithelia at ALI (Figure 1E). The data shown are representative images from one donor cell culture; comparable results were observed in an independent donor cell culture treated similarly. Scale bars = 37 µm.

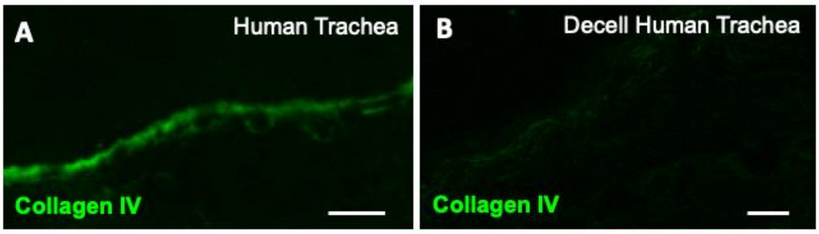

Supplementary Figure S4: Immunofluorescence staining of sections of native human airway (A) and decellularized trachea (B). Collagen IV (green) was observed in native human airway. However, collagen IV reactivity is lost following decellularization, indicating a disruption of the basement membrane during this process. Scale bars = 50 µm.

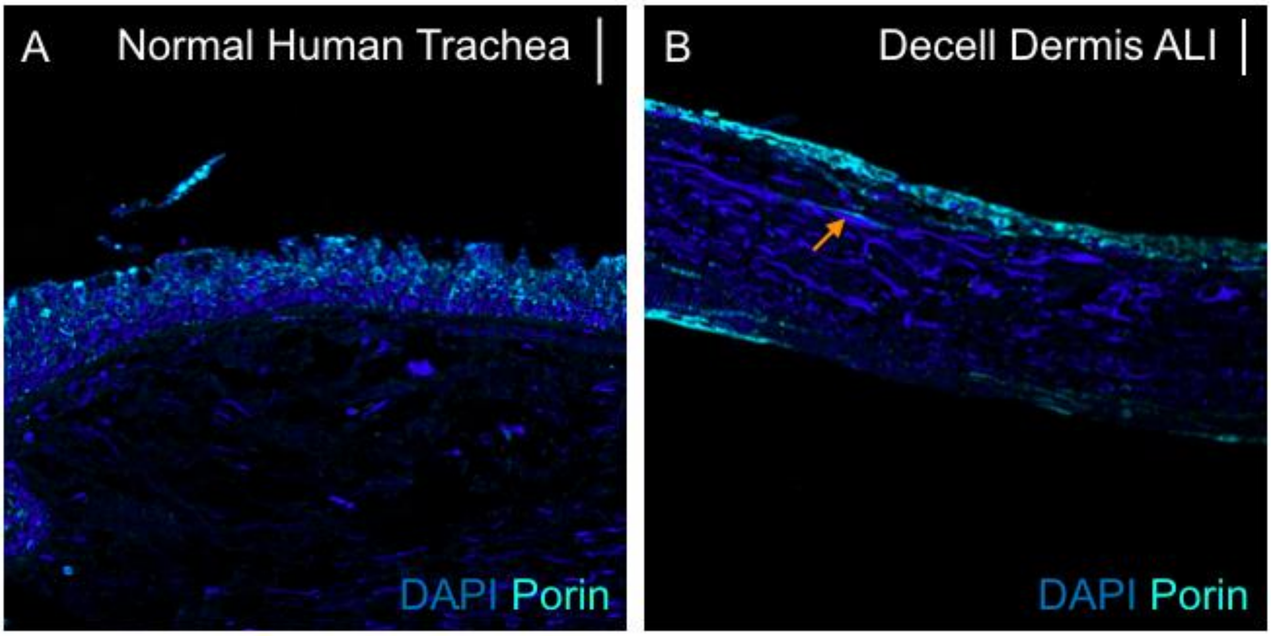

Supplementary Figure S5: Porin immunofluorescence staining (cyan) in a decellularized dermis-based scaffold seeded with airway epithelial cells and lung fibroblasts after 21 days at ALI.

Sections were counterstained using DAPI (nuclei; blue). Scale bars = 50 µm.
