# Supplementary material for: Bioengineered airway epithelial grafts with mucociliary function based on collagen IV- and laminin-containing extracellular matrix scaffolds
Source: Eur Respir J. 2020 Jun 18;55(6):1901200. doi: 10.1183/13993003.01200-2019 (PMC7301290; doi:10.1183/13993003.01200-2019)
Supplement: Supplementary file 3 [file ERJ-01200-2019.Shareable.pdf]

ORIGINAL ARTICLE
BASIC SCIENCE

SHAREABLE PDF

Bioengineered airway epithelial grafts with mucociliary function based on collagen IV- and laminin-containing extracellular matrix scaffolds

Nick J.I. Hamilton [1,2,6], Dani Do Hyang Lee [3], Kate H.C. Gowers [1], Colin R. Butler [1], Elizabeth F. Maughan [1], Benjamin Jevans[4], Jessica C. Orr [1], Conor J. McCann [4], Alan J. Burns [4], Sheila MacNeil[5], Martin A. Birchall [2], Christopher O'Callaghan [3], Robert E. Hynds [1] and Sam M. Janes [1,6]

Affiliations: [1]Lungs for Living Research Centre, UCL Respiratory, University College London, London, UK. [2]UCL Ear Institute, The Royal National Throat Nose and Ear Hospital, London, UK. [3]Respiratory, Critical Care and Anaesthesia, UCL Great Ormond Street Institute of Child Health, London, UK. [4]Stem Cell and Regenerative Medicine, Birth Defects Research Centre, UCL Great Ormond Street Institute of Child Health, London, UK. [5]Dept of Materials and Science Engineering, The Kroto Research Institute, North Campus, University of Sheffield, Sheffield, UK. [6]Nick J.I. Hamilton and Sam M. Janes are joint-senior authors.

Correspondence: Nick J.I. Hamilton, UCL Ear Institute, University College London, 332 Grays Inn Road, London, WC1X 8EE, U.K. E-mail: nick.hamilton@ucl.ac.uk

@ERSpublications
Collagen IV- and laminin-rich decellularised dermis scaffolds support a mucociliary airway epithelial graft but *in vivo* transplantation in pre-clinical models is challenging http://bit.ly/2IdQp5d

Cite this article as: Hamilton NJI, Lee DDH, Gowers KHC, *et al*. Bioengineered airway epithelial grafts with mucociliary function based on collagen IV- and laminin-containing extracellular matrix scaffolds. *Eur Respir J* 2020; 55: 1901200 [https://doi.org/10.1183/13993003.01200-2019].

This single-page version can be shared freely online.

ABSTRACT Current methods to replace damaged upper airway epithelium with exogenous cells are limited. Existing strategies use grafts that lack mucociliary function, leading to infection and the retention of secretions and keratin debris. Strategies that regenerate airway epithelium with mucociliary function are clearly desirable and would enable new treatments for complex airway disease.

Here, we investigated the influence of the extracellular matrix (ECM) on airway epithelial cell adherence, proliferation and mucociliary function in the context of bioengineered mucosal grafts. *In vitro*, primary human bronchial epithelial cells (HBECs) adhered most readily to collagen IV. Biological, biomimetic and synthetic scaffolds were compared in terms of their ECM protein content and airway epithelial cell adherence.

Collagen IV and laminin were preserved on the surface of decellularised dermis and epithelial cell attachment to decellularised dermis was greater than to the biomimetic or synthetic alternatives tested. Blocking epithelial integrin α2 led to decreased adherence to collagen IV and to decellularised dermis scaffolds. At air–liquid interface (ALI), bronchial epithelial cells cultured on decellularised dermis scaffolds formed a differentiated respiratory epithelium with mucociliary function. Using *in vivo* chick chorioallantoic membrane (CAM), rabbit airway and immunocompromised mouse models, we showed short-term preservation of the cell layer following transplantation.

Our results demonstrate the feasibility of generating HBEC grafts on clinically applicable decellularised dermis scaffolds and identify matrix proteins and integrins important for this process. The long-term survivability of pre-differentiated epithelia and the relative merits of this approach against transplanting basal cells should be assessed further in pre-clinical airway transplantation models.

Copyright ©ERS 2020. This version is distributed under the terms of the Creative Commons Attribution Licence 4.0.
